# Enhanced Detection of Tuberculous Mycobacteria in Animal Tissues Using a Semi-Nested Probe-Based Real-Time PCR

**DOI:** 10.1371/journal.pone.0081337

**Published:** 2013-11-21

**Authors:** Pedro Costa, Ana S. Ferreira, Ana Amaro, Teresa Albuquerque, Ana Botelho, Isabel Couto, Mónica V. Cunha, Miguel Viveiros, João Inácio

**Affiliations:** 1 Unidade Estratégica de Investigação e Serviços em Produção e Saúde Animal, Instituto Nacional de Investigação Agrária e Veterinária, I.P., (INIAV, I.P.), Lisboa, Portugal; 2 Grupo de Micobactérias, Unidade de Ensino e Investigação de Microbiologia Médica, Instituto de Higiene e Medicina Tropical, Universidade Nova de Lisboa, Lisboa, Portugal; 3 Instituto de Ciências Biomédicas de Abel Salazar, Universidade do Porto, Porto, Portugal; 4 Centro de Recursos Microbiológicos (CREM), Universidade Nova de Lisboa, Lisboa, Portugal; 5 Centro de Malária e Outras Doenças Tropicais, Instituto de Higiene e Medicina Tropical, Universidade Nova de Lisboa, Lisboa, Portugal; University College Dublin, Ireland

## Abstract

Bovine tuberculosis has been tackled for decades by costly eradication programs in most developed countries, involving the laboratory testing of tissue samples from allegedly infected animals for detection of Mycobacterium tuberculosis complex (MTC) members, namely *Mycobacterium bovis*. Definitive diagnosis is usually achieved by bacteriological culture, which may take up to 6–12 weeks, during which the suspect animal carcass and herd are under sanitary arrest. In this work, a user-friendly DNA extraction protocol adapted for tissues was coupled with an IS*6110*-targeted semi-nested duplex real-time PCR assay to enhance the direct detection of MTC bacteria in animal specimens, reducing the time to achieve a diagnosis and, thus, potentially limiting the herd restriction period. The duplex use of a novel *β-actin* gene targeted probe, with complementary targets in most mammals, allowed the assessment of amplification inhibitors in the tissue samples. The assay was evaluated with a group of 128 fresh tissue specimens collected from bovines, wild boars, deer and foxes. *Mycobacterium bovis* was cultured from 57 of these samples. Overall, the full test performance corresponds to a diagnostic sensitivity and specificity of 98.2% (CI_P95%_ 89.4–99.9%) and 88.7% (CI_P95%_ 78.5–94.7%), respectively. An observed kappa coefficient was estimated in 0.859 (CI_*P95%*_ 0.771–0.948) for the overall agreement between the semi-nested PCR assay and the bacteriological culture. Considering only bovine samples (n = 69), the diagnostic sensitivity and specificity were estimated in 100% (CI_P95%_ 84.0–100%) and 97.7% (CI_P95%_ 86.2–99.9%), respectively. Eight negative culture samples exhibiting TB-like lesions were detected by the semi-nested real-time PCR, thus emphasizing the increased potential of this molecular approach to detect MTC-infected animal tissues. This novel IS*6110*-targeted assay allows the fast detection of tuberculous mycobacteria in animal specimens with very high sensitivity and specificity, being amenable and cost effective for use in the routine veterinary diagnostic laboratory with further automation possibilities.

## Introduction

Tuberculosis (TB) is a leading cause of morbidity and mortality in the world, also affecting a wide range of animal species, particularly livestock, in both developed and developing countries. The disease is caused by tuberculous mycobacteria belonging to the Mycobacterium tuberculosis complex (MTC). This complex consists of several closely-related pathogenic species, namely *M. tuberculosis*, the main agent of human TB, and, amongst others, *M. bovis* and *M. caprae* that are primary agents of bovine and caprine TB, respectively [1]. These species are genetically very similar but may differ in host preference and epidemiological characteristics [2]. *Mycobacterium bovis* and *M. caprae* also represent a high potential for zoonotic transmission to humans [3–5], with evidence of possible person-to-person transmission [6]. However, the main routes of transmission are the contact with infected animals and ingestion of unpasteurized dairy products. These zoonotic MTC species may be responsible for up to 7.2% and 15% of human TB cases in industrialized and developing countries, respectively [7]. Rapid and reliable laboratory tests for the direct detection of tuberculous mycobacteria in biological samples are in high demand, in both human health and veterinary settings, and are crucial for an improved TB control. 

In most developed countries, bovine tuberculosis has been tackled during the last decades by costly eradication programs, involving the culling of reactor animals and laboratory testing of suspect samples for the definitive confirmation of the presence of MTC. Presently, the detection of MTC bacteria in animal tissues is mainly based in lengthy and cumbersome conventional methods, involving the examination of Ziehl-Neelsen stained smears, histopathology and culture in selective media, followed by biochemical or molecular identification of typical mycobacteria colonies. The microscopic identification of acid-fast mycobacteria is non-specific and highly insensitive, particularly in the case of paucibacillary forms of TB. Culture remains the gold-standard method to confirm TB infection but requires several weeks to obtain positive results due to the extremely fastidious growth of tuberculous mycobacteria. In spite of the significant advances in the development of novel molecular diagnostic assays towards a faster and accurate detection of MTC in human samples, only a few assays have been described for detecting these agents in animal tissues, particularly in fresh tissues from livestock [8-12]. Most of these molecular approaches are PCR-based and target specific polymorphisms, insertion sequences, and the so-called regions of difference in the genome of MTC members [9,10,13-18]. Nevertheless, most of the amplification-based assays described for detecting MTC nucleic acids directly in fresh or formalin-fixed paraffin-embedded tissues only yield a moderate sensitivity, usually up to 75%, particularly when testing tissues without the characteristic lesions or detectable acid-fast bacilli [8-10,15,19]. This limitation is mostly related to the inefficiency of mycobacterial DNA extraction procedures from affected animal tissues, especially those exhibiting strong fibrosis, calcification, and with no histological evidence of acid-fast bacteria [8,19]. The use of immunomagnetic separation approaches to concentrate mycobacteria cells from animal tissues prior to DNA extraction may enhance PCR sensitivities [20,21]. Nevertheless, these approaches usually involve more experimental steps and expensive equipment and consumables not readily available in veterinary diagnostic settings.

In the present work we have developed a novel and simple *Taqman*-based semi-nested real-time PCR assay yielding extremely high sensitivity and specificity for the direct detection of tuberculous mycobacteria in fresh animal tissues, namely of bovine origin, capable of being introduced in routine diagnostic veterinary laboratories.

## Materials and Methods

### Bacterial strains

Reference strains and clinical isolates of MTC, non-MTC mycobacteria and non-mycobacterial species, maintained at the Portuguese reference laboratory for animal diseases (INIAV, IP), were used for optimization of PCR assays (Table 1). The identification of each isolate was based on standard methodologies [22]. MTC isolates were identified to the species level by PCR-restriction endonuclease analysis of the *gyrB* gene [23–25] and hybridization with species-specific probes [26].

**Table 1:** Bacterial reference strains and clinical isolates whose cultures were used in the present study for the evaluation of specificity of the amplification assays and respective results.

**Species**	**Reference strains/Isolates**	**Presence of IS*6110*^*1*^**
*Mycobacterium tuberculosis*	ATCC 25177; LNIV 9605	+
*M. bovis*	AN5; ATCC 27291 (BCG); LNIV 13027; 5530/0/05; 11265; 7230/4; 14421/2; 24497/6; 8855; 5889; 10044; 14577; 13280/6; 13280/4; 34875; and 20564	+
*M. caprae*	LNIV 17320; 4958/0/05; 8403; 15244; and 20752	+
*M. avium* subsp. *avium*	ATCC 25291	-
*M. avium* subsp. *hominissuis*	LNIV 23063/4	-
*M. avium* subsp. *paratuberculosis*	LNIV 39888	-
*M. scrofulaceum*	LNIV 31389	-
*Acinetobacter baumannii*	LNIV 1628/12	-
*Arcanobacterium pyogenes*	VLA 1884	-
*Bacillus licheniformis*	VLA 1831	-
*Corynebacterium striatum*	LNIV 12352	-
*Enterobacter amnigenus*	LNIV 6050/II	-
*Klebsiella pneumonia*	VLA 1643	-
*Listeria monocytogenes*	VLA 1774	-
*Proteus mirabilis*	LNIV 2269/II	-
*Pseudomonas aeruginosa*	VLA 67	-
*Salmonella Dublin*	VLA 1272	-
*Staphylococcus aureus*	VLA 1032	-
*Streptococcus agalactiae*	VLA 33	-
*Yersinia enterocolitica*	VLA 1884	-

ATCC, American Type Culture Collection, USA; LNIV, Laboratório Nacional de Investigação Veterinária (*currently INIAV, IP*), Lisbon, Portugal; VLA, Veterinary Laboratory Agency, UK; **^*1*^**Amplification (+) or no amplification (-) of IS*6110* element using the real-time PCR assay with P_IS*6110* TaqMan probe and respective flanking primers F_IS*6110* and R_IS*6110*.

#### Tissue samples

One hundred and twenty eight animal lymph nodes, liver, spleen or lung tissue samples (69 bovines, 35 wild boars, 15 deer and 9 foxes) were used in this work (Table 2). No animals were sacrificed for the purposes of this specific study. None of the authors were responsible for the death of any animals and samples were originally collected for purposes other than research, namely: (i) bovine samples were collected from animals clinically suspected of having TB, e.g. by a positive reaction in either the single intradermal comparative tuberculin test or the gamma-interferon test, or TB-like lesions detected during routine abattoir inspection, and were submitted to routine control testing under the governmental Portuguese eradication scheme for bovine tuberculosis, approved in 1992 by the European Union (Council Decision 92/299/CE) and, since 2001, cofinanced by the European Union (in the framework of Council Directive 64/432, as amended) [27]; and (ii) TB suspect samples from wild boar, deer and fox, were sent to INIAV reference laboratories following gross pathological evaluation performed in the field by local veterinarians in hunting activities or predator control actions legally authorized by the "*Instituto da Conservação da Natureza e das Florestas*" (the Portuguese National Forest Authority), that grants permits for those hunting actions and for the respective hunters. Samples were submitted during the fourth trimester of 2011 to the pathology and bacteriology laboratories of INIAV and analysed using routine histological and culture-based methods, according to the OIE standard procedures [22] (Table 2). Tissues selected for bacteriological analysis were homogenized using a pestle and mortar, followed by decontamination with 4% sodium hydroxide. After neutralization with 10% hydrochloric acid, the macerated suspensions were divided into equal parts. One part was maintained at -80 °C until further processing for molecular analysis and the other was centrifuged and the sediment inoculated into BACTEC 9000 liquid media and Lowenstein-Jensen with pyruvate and Stonebrink solid media. Inoculated media were incubated for a minimum of eight weeks at 37 °C. Heat-killed culture supernatants were kept at -20 °C until the molecular identification of the isolates. Species identification of presumptive mycobacteria isolates was based on the restriction endonuclease analysis with *Rsa*I and *Sac*II of the PCR-amplified *gyr*B gene, or using the commercial reverse hybridization assays INNO-LiPA Mycobacteria (Innogenetics, Belgium) or GenoType *Mycobacterium* (Hain diagnostics, Germany), following the manufacturer’s instructions [23-25]. 

**Table 2:** Typology of tissue samples used in this study (n = 128) and respective results of the histological, bacteriological and semi-nested duplex real-time PCR analyses.

**Sample typology**	**Number of tissue samples**	**Origin of tissues**	**Presence of lesions^*1*^**	**Bacteriological analysis^*2*^**	**Nested real-time PCR^*3*^**
	23	Bovine	+	*M. bovis*	23
I	19	Wild boar	+	*M. bovis*	18
	12	Deer	+	*M. bovis*	12
II	3	Bovine	-	*M. bovis*	3
	39	Bovine	-	-	0
III	4	Wild boar	-	-	0
	3	Deer	-	-	0
IV	2	Bovine	-	Non-MTC	0
	3	Wild boar	-	Non-MTC	0
	1	Bovine	+	-	0
V	5	Wild boar	+	-	3
	6	Fox	+	-	0
	1	Bovine	+	Non-MTC	1
VI	4	Wild boar	+	Non-MTC	2
	3	Fox	+	Non-MTC	2

***^1^***Presence of lesions compatible with tuberculosis; **^*2*^**Detection of *M. bovis* or other non-MTC mycobacteria by culture of tissue samples; **^*3*^**Number of samples for which the IS*6110* element was amplified by the nested real-time PCR assay.

### Spiked tissue samples

A lymph node tissue from a slaughtered bovine known to be free of TB, as confirmed by culture and histopathology, was used for testing as a spiked sample that was homogenized as described above. Eight aliquots containing 0.9 ml of tissue macerate were spiked with 0.1 ml of ten-fold dilutions of a suspension of *M. tuberculosis* H37Ra (ATCC 25177) cells. Estimated concentrations of mycobacteria in spiked macerate samples ranged from 10^7^ to 10° cells/ml. An additional tube was spiked with 0.1 ml PBS buffer and used as negative control. Spiked samples were stored at - 20 °C until processing for DNA extraction. 

### DNA extraction from cultures

DNA extraction from cultures grown in liquid media was achieved by a combined bead beating and enzymatic extraction method described elsewhere [28]. Briefly, 0.5 ml bacterial culture were washed in PBS buffer and heat inactivated at 100 °C for 15 min. Tubes containing zirconium beads were used to resuspend the culture pellet in lysis buffer (0.4 M NaCl, 40 mM Tris-HCl, pH 8, 2 mM EDTA, 0.6% SDS, 0.034 mg/ml proteinase K) for mechanical disruption in the FastPrep FP120 Bio101 (Savant Instruments, Inc., Holbrook, NY) at 6.5 ms^-1^ for 45 s, and then incubated overnight at 37 °C, followed by standard phenol–chloroform puriﬁcation and ethanol precipitation of DNA. DNA concentration and purity were estimated by measuring the absorbance at 260 nm (A260) and by A260 ⁄A280 and A260/A230 ratios, respectively, using a NanoDrop 1000 spectrophotometer (NanoDrop). Genomic DNA suspensions were stored at - 20 °C until further use.

### DNA extraction from tissue macerates and spiked samples

Four hundred and fifty microliters of tissue suspensions were transferred to screw-cap microcentrifuge tubes and inactivated in a water bath at 100 °C for 5 minutes. Samples were centrifuged at 14000 rpm for 2 min, the supernatant rejected and 80 µl of PBS and an equivalent volume of 100 µl of zirconium beads were added to the tubes. After mechanical disruption in a FastPrep FP120 Bio101 bead shaker (Savant Instruments Inc., , Holbrook, NY) at 6.5 msec^-1^ for 45 seconds, repeated three times, the suspensions were cooled on ice for 15 minutes. DNA extraction was carried out using the tissue protocol of the QIAamp DNA Mini Kit (Qiagen) according to the manufacturer's instructions. The resulting genomic DNA suspensions were stored at - 20 °C until further use. Stock DNA suspensions were diluted ten times in distilled water before its use as template for PCR assays.

### Design of TaqMan probes and flanking primers

**Figure 1:**
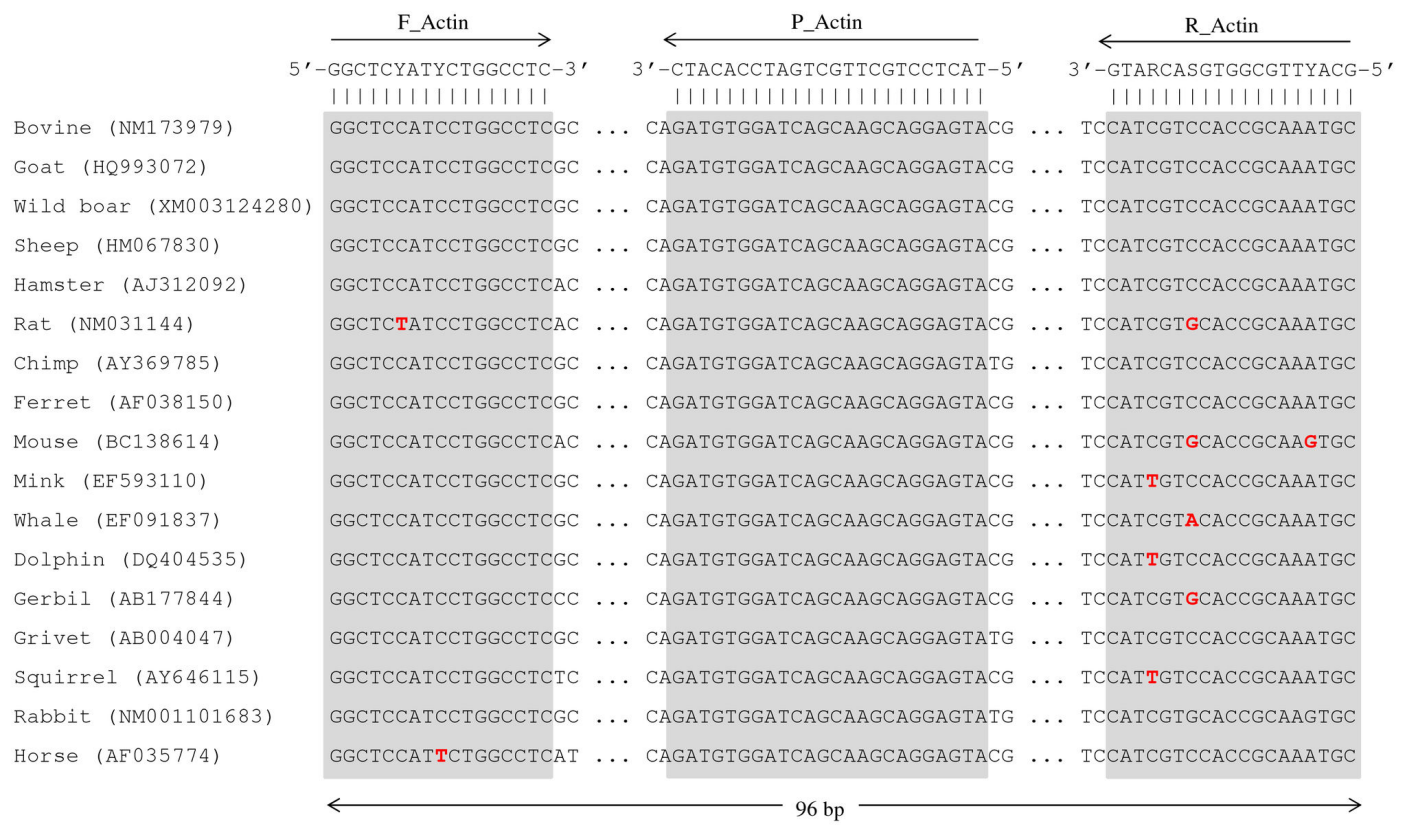
Complementary targets of the mammals β-actin gene targeted probe and flanking primers. Partial alignment of β-actin gene sequences of several mammal species showing the complementary targets of the P_Actin TaqMan probe and respective flanking degenerated primers (F_Actin and R_Actin) (gray boxes). The GenBank access numbers from which the partial sequences were retrieved are indicated for each species inside parenthesis. Mismatches in relation to consensus sequence are highlighted in red.

**Figure 2:**
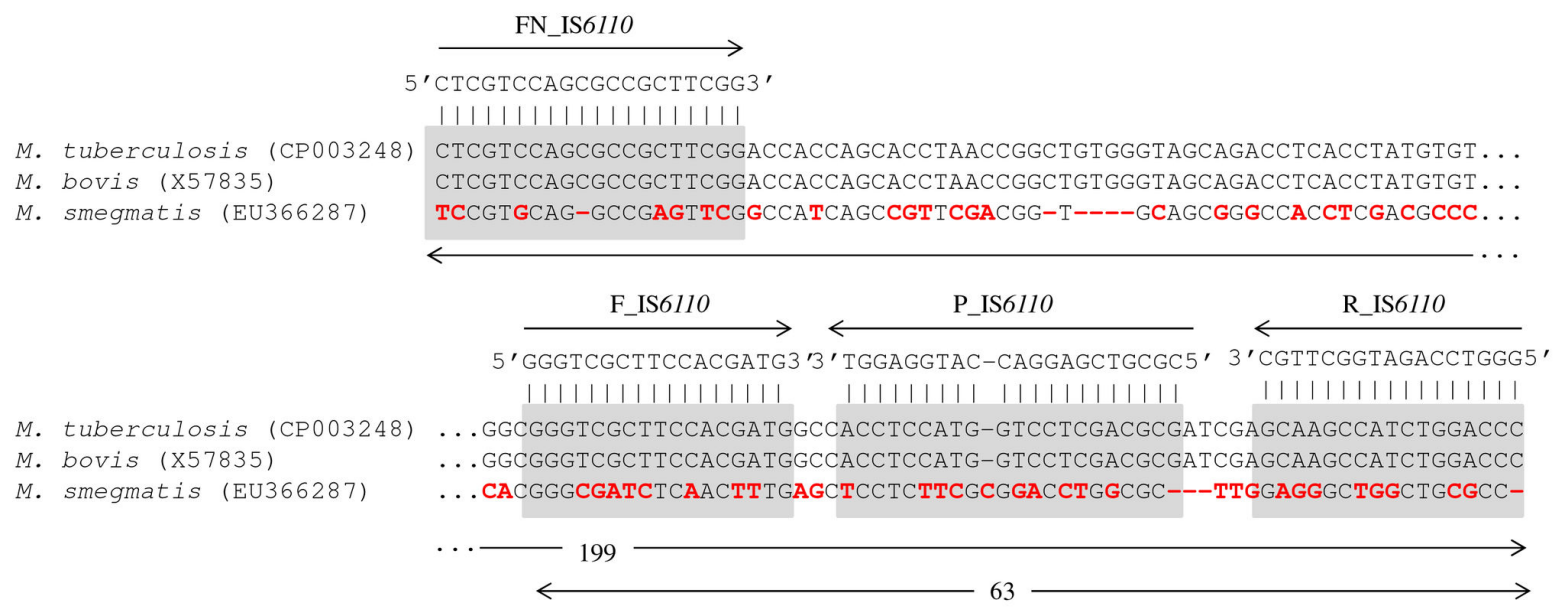
Complementary targets of the MTC-specific IS*6110*-targeted probe and flanking primers. Partial alignment of the IS*6110* sequence of *Mycobacterium tuberculosis* complex members with other IS*6110*-like sequence recently found in *M. smegmatis* (GenBank access numbers are indicated inside parenthesis). The complementary targets of the P_IS*6110* *TaqMan* probe and flanking primers (FN_IS*6110*, F_IS*6110* and R_IS*6110*) are indicated (gray boxes). Mismatches in sequences are highlighted in red.

Sequences of IS*6110* from MTC members and *β-actin* gene from a wide range of mammal species were retrieved from NCBI-GenBank. Comparative analysis of these two sets of sequences was achieved through sequence alignment using the CLUSTAL X v2.0 software [29]. Complementary regions for a *β-actin* gene-targeted mammals-universal *TaqMan* probe and flanking degenerated primers were found after visual inspection of the respective alignments (Table 3; Figure 1). The amplification of *β-actin* gene served as control to detect inhibition of the PCR reactions when using DNA extracted from tissues as template. The IS*6110*-targeted probe (P_IS*6110*) and respective flanking primers (F_IS*6110* and R_IS*6110*) were retrieved from the study of Restrepo and colleagues [30] (Table 3; Figure 2). An additional IS*6110*-targeted forward primer (FN_IS*6110*) for use in a semi-nested PCR was designed (Table 3; Figure 2). Probes and primers specificities were tested *in silico* using the BLAST tools from NCBI-GenBank. All probes and primers targeting IS*6110* and *β-actin* gene were synthesized by MWG Biotech (Germany).

**Table 3:** Sequences of primers and probes used in this study.

**Primer/Probe**	**Sequence (5´-3´)**	**Complementary target**
F_Actin	GGC TCY ATY CTG GCC TC	*β-actin* gene of mammals
R_Actin	GCA YTT GCG GTG SAC RAT G	
P_Actin**^*1*^**	Cy5.5-TAC TCC TGC TTG CTG ATC CAC ATC-BHQ2	
F_IS*6110*	GGG TCG CTT CCA CGA TG	IS*6110* element of MTC species
FN_IS*6110*	CTC GTC CAG CGC CGC TTC GG	
R_IS*6110*	GGG TCC AGA TGG CTT GC	
P_IS*6110 * **^*2*^**	FAM-CGC GTC GAG GAC CAT GGA GGT-BHQ1	

***^1^***Probe labeled with Cy5.5 fluorophore and BHQ-2 quencher; **^*2*^**Probe labeled with carboxyfluorescein (FAM) fluorophore and BHQ-1 quencher

### Amplification assays

The semi-nested real-time PCR amplification assay using DNA extracted from tissue samples as template consisted of two steps: (i) a first standard PCR using primers FN_IS*6110* and R_IS*6110*; and (ii) a second duplex real-time PCR using the previous amplification product as template and a mixture of IS*6110* and *β-actin* gene targeted *TaqMan* probes (P_IS*6110* and P_Actin, respectively) and the corresponding flanking primers (F_IS*6110*/R_IS*6110* and F_Actin/R_Actin, respectively) (Table 3). IS*6110*-targeted amplification reactions were previously optimized using DNA extracted from pure cultures as template. The first standard PCR step amplifies an MTC-specific 199 bp fragment of the IS*6110* (Figure 2). PCR reactions were carried out in a final volume of 25 μl containing 400 μM of deoxynucleotide triphosphates (Promega), 1 U of *Taq* DNA polymerase (Promega), 3.5 mM of MgCl_2_ (Promega), 0.8 μM of each primer (FN_IS*6110* and R_IS*6110*), DNase free water (GIBCO) and 5 μl of extracted DNA template. Amplification was performed in a C1000 thermocycler (Bio-Rad) using the following program: initial denaturation step at 95 °C for 10 min, 45 cycles of 30 sec at 95 °C, 30 sec at 65 °C, 30 sec at 72 °C, and a final extension step of 10 min at 72 °C. The amplified products were stored at 4 °C until electrophoresis analyses in a 2% agarose gel or directly used for the second duplex real-time PCR step. Real-time PCR reactions were carried out in a total volume of 20 µl containing 1× SSO Fast Super Mix (Bio-Rad), 0.4 μM of each primer (F_IS*6110*, R_IS*6110*, F_Actin and R_Actin), 0.15 μM of each TaqMan probe (P_IS*6110* and P_Actin), DNase free water (GIBCO) and 5 µl of the previous PCR products. The thermal cycling conditions were as follows: 1 cycle at 95 °C for 2 min, followed by 45 cycles at 95 °C for 5 s and 60 °C for 10 s. All samples that probed positive for IS*6110* were retested for confirmation. Thermal cycling, fluorescent data collection, and data analysis were performed in a CFX96 (Bio-Rad) detection system real-time PCR instrument, according to the manufacturer’s instructions.

### Analytical specificity and sensitivity

To investigate whether the *IS6110*-targeted real-time PCR assay specifically amplifies DNA from MTC members, MTC and non-MTC mycobacterial isolates were tested, as well as other clinically relevant bacteria (Table 1). To estimate the detection threshold of the assay (analytical sensitivity), a standard curve was constructed using 10-fold serial dilutions of DNA extracted from *M. tuberculosis* H37Ra (ATCC 25177) as template. Each template was run in triplicate. The end-point corresponded to the dilution at which the assay could not detect the target in at least one of the replicates. 

### Detection limit of the IS*6110*-targeted semi-nested real time PCR assay

The detection limit of the semi-nested duplex real time PCR assay was assessed using the serially spiked tissue macerate samples. Each template was tested in triplicate.

### Diagnostic specificity and sensitivity

The tissue samples were stratified in six typologies (Table 2): I - lesions compatible with tuberculosis are present and *M. bovis* was cultured from the samples (n = 54); II- lesions are not present but *M. bovis* was cultured from samples (n = 3); III- absence of any lesions and mycobacteria were not cultured from samples (n = 46); IV- absence of lesions but non-MTC mycobacteria were cultured from samples (n = 5); V- lesions compatible with tuberculosis are present but mycobacteria were not cultured (n = 12); and VI - lesions are present and non-MTC mycobacteria were cultured from samples (n = 8). The culture of *M. bovis* from tissue samples was used as gold-standard reference method for the computation of the diagnostic sensitivity and specificity of the semi-nested real-time PCR assay. Overall diagnostic sensitivity, specificity and positive (PPV) and negative (NPV) predictive values were computed using all culture positive (types I + II; n = 57) and negative (types III, IV, V and VI; n = 71) tissue samples (Table 2). Additionally, these parameters were also computed using only the bovine culture positive (n = 26) and negative (n = 43) samples (Table 2). Although sample types V and VI were also culture negative for *M. bovis*, lesions compatible with the disease were found. For the computation of the kappa coefficient, for measuring the agreement between the gold-standard method of bacteriological culture and the IS*6110*-targeted semi-nested PCR assay, all tissue samples were used (types I - VI, n = 128). Sensitivity, specificity, PPV, NPV and kappa coefficient, with confidence intervals, were computed using the clinical research calculators of the online VassarStats software (http://vassarstats.net).

## Results

### Design of probes and primers

A novel set of *β-actin* gene-targeted *TaqMan* probe and respective flanking primers was designed (Table 3, Figure 1). *In silico* analysis using the BLAST suite of NCBI-GenBank confirmed that the complementary regions of this probe, and primers, are widespread amongst most mammal species, including livestock animals. *In silico* analysis also confirmed that the complementary regions of the IS*6110*-targeted probes and primers were only present in IS*6110* sequences of MTC members (Table 3, Figure 2). 

#### Analytical specificity and sensitivity

**Figure 3:**
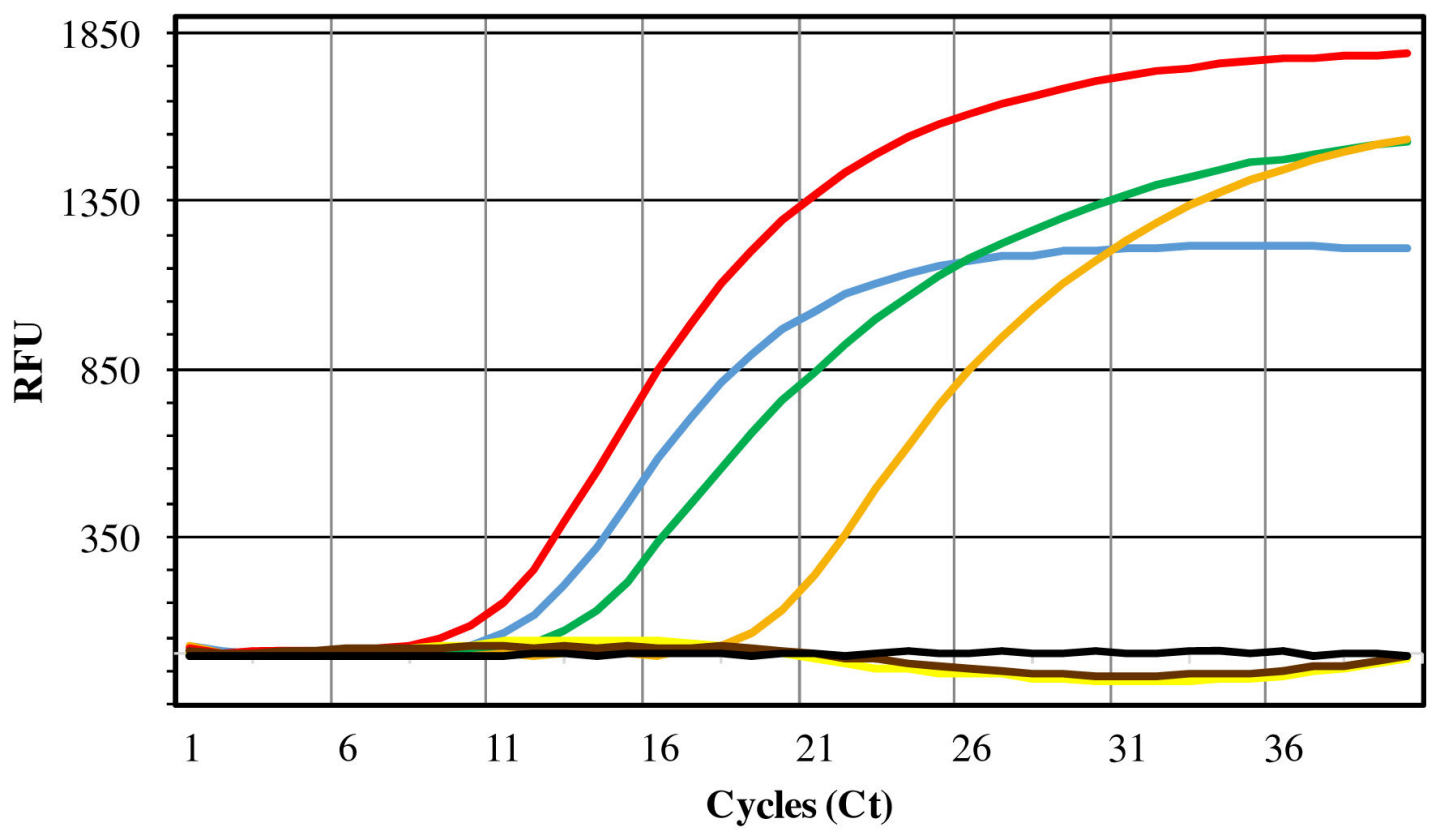
IS*6110*-targeted real-time PCR amplification curves obtained in specificity tests. Illustration of the MTC-specific amplification curves obtained in FAM channel during evaluation of the analytical specificity of the IS*6110*-targeted real-time PCR step. All MTC cultures yielded amplification curves: *M. tuberculosis* ATCC 25177 (blue line), *M. bovis* LNIV 13027 (green line), *M. bovis* ATCC 27291 (orange line) and *M. caprae* LNIV 17320 (red line). Other bacteria yielded negative results of amplification: *M. avium* subsp. *avium* ATCC 25291 (yellow line) and *M. avium* subsp. *paratuberculosis* LNIV 39888 (brown line). No amplification was detected in non-template negative control (black line). RFU - Relative Fluorescence Units.

The real-time PCR assay with the IS*6110*-targeted probe (P_IS*6110*) and flanking primers (F_IS*6110* and R_IS*6110*) yielded amplification products only when using DNA extracted from MTC members as template (Table 1, Figure 3). No non-specific results were obtained with members of the *Mycobacterium avium* complex or with strains belonging to other diverse bacterial species. The minimum detection threshold (analytical sensitivity) of this assay was estimated by the construction of a reference curve with serially-diluted suspensions of DNA from *M. tuberculosis* H37Ra. The analytical sensitivity was estimated in 0.3 fg/µL of *M. tuberculosis* genomic DNA. A linear relationship between the logarithm of the starting concentration of DNA and the amplification Ct values was obtained, with a -3.222 slope, a Ct = 35 interception in the minimum threshold (0.3 fg/µL) and an R^2^ = 0.999.

#### Spiked samples

**Figure 4:**
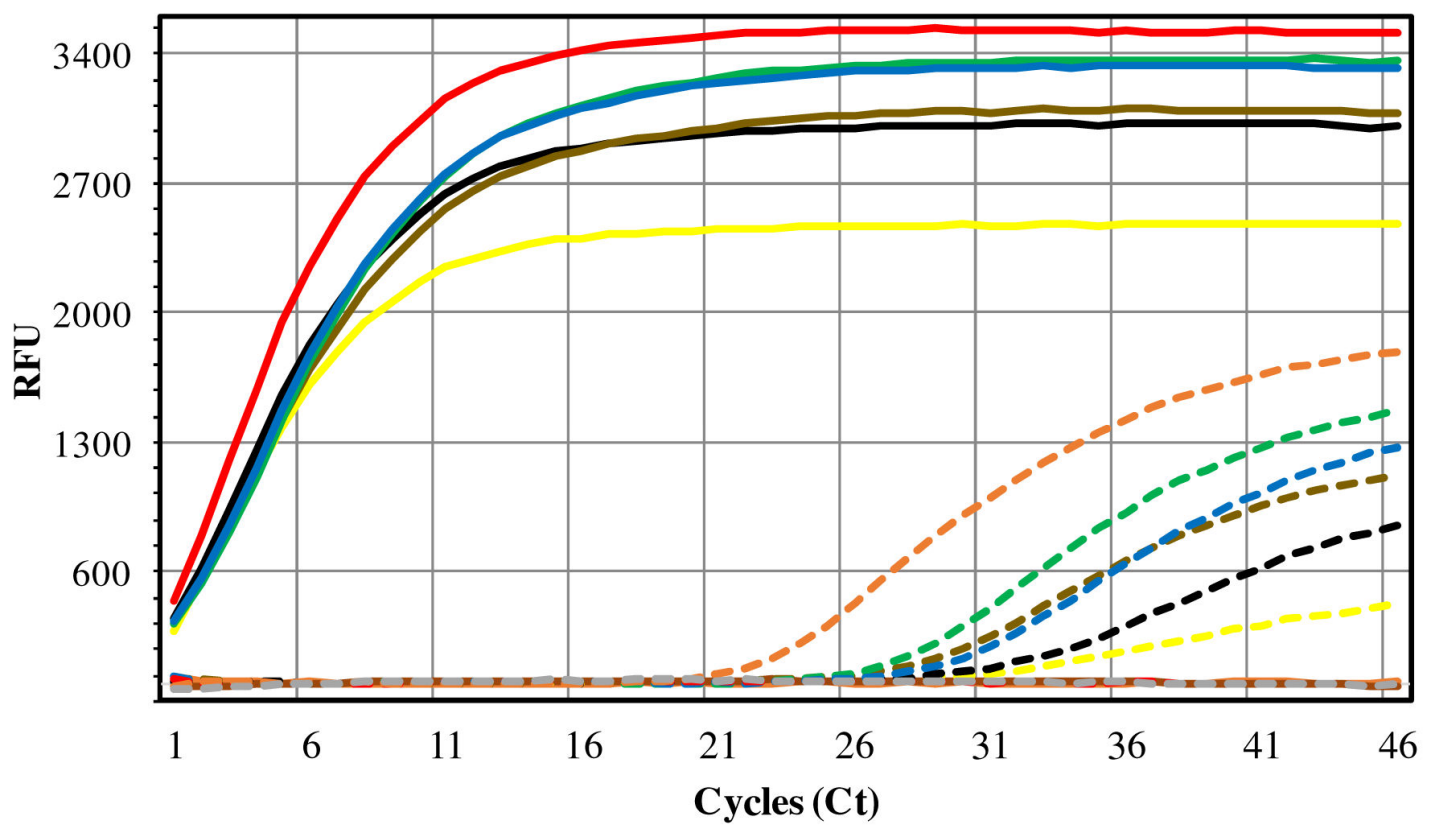
Testing of spiked samples with the semi-nested duplex real-time PCR assay. The analytical sensitivity of the assay was estimated through the use of solutions of DNA extracted from MTC-free tissue samples spiked with serially diluted cellular suspensions of *M. tuberculosis* ATCC 25177 (ranging from 10^7^ to 10° cells/ml of tissue homogenate). The figure illustrates the IS*6110* (solid lines) and mammal *β-actin* gene (dashed lines) targeted amplification curves obtained in FAM and Cy5.5 channels, respectively, for the several dilutions: 10^7^ (yellow line), 10^5^ (black line), 10^3^ (brown line), 10^1^ (green line) and 10° (blue line) cells. Positive control: *M. tuberculosis* ATCC 25177 culture (red line); Negative controls: unspiked tissue homogenate (orange line) and non-template control (grey line). RFU - Relative Fluorescence Units.

Tissue samples spiked with serially-diluted suspensions of *M. tuberculosis* cells were used for assessing the detection limit of the *β-actin* and IS*6110*-targeted semi-nested duplex real-time PCR assay. The detection threshold was estimated to be one mycobacteria per ml of tissue macerate (Figure 4). The IS*6110* amplification curves usually harboured a very low Ct (

< 3), meaning that the first standard PCR step yielded abundant amplification products easily detected by the following real time PCR step . The co-amplification of the bovine *β-actin* gene showed that no apparent inhibition of the real-time PCR step occurred due to the presence of inhibitor components of tissues or excess of bovine DNA, a significant result for the type of specimens used (animal tissues), confirming the efficiency of the extraction and purification procedures used in this work (Figure 4).

#### Diagnostic specificity and sensitivity

The results of the semi-nested duplex real-time PCR assays using DNA extracted from tissue samples are summarized in Table 2. All but one tissue sample from which *M. bovis* was cultured (types I and II) yielded positive amplification results, corresponding to a diagnostic sensitivity of 98.2% (CI_P95%_ 89.4-99.9%). Noteworthy, when a subset of these TB culture positive samples was preliminary tested using only the second step of the amplification assay (without the first conventional PCR step), the diagnostic sensitivity was only 40%. Eight DNA-positive tissues were obtained among the 71 *M. bovis* culture negative samples (types III, IV, V and VI), yielding a diagnostic specificity of 88.7% (CI_P95%_ 78.5-94.7%). Negative controls run with batches of samples did not identify any cross-contaminating DNA. The positive and negative predictive values were estimated in 87.5% (CI_P95%_ 76.3-94.1%) and 98.4% (CI_P95%_ 90.1-99.9%), respectively, for a prevalence of 44.5% *M. bovis* culture positive samples among all samples analysed. Considering only the bovine samples, the diagnostic sensitivity and specificity of the amplification assay were estimated in 100% (CI_P95%_ 84.0-100%) and 97.7% (CI_P95%_ 86.2-99.9%), respectively, while PPV and NPV were estimated in 96.3% (CI_P95%_ 79.1-99.8%) and 100% (CI_P95%_ 89.6-100%), respectively, for a prevalence of 37.7% *M. bovis* culture positive samples.

No DNA-positive samples were obtained from samples without TB-compatible lesions and *M. bovis* isolation (types III and IV) (Table 2). However, eight DNA-positive samples were detected among tissues that were *M. bovis* culture-negative but that harboured TB-compatible lesions (sample types V and VI) (Table 2). 

An observed kappa coefficient was estimated in 0.859 (CI_P95%_ 0.771-0.948) for the overall agreement between the results obtained by the direct application of the IS*6110*-targeted semi-nested PCR assay to fresh tissue samples and the results obtained from the gold-standard bacteriological culture. The direct detection procedure allowed the detection of MTC infected samples in less than 6 hours while the conventional culture takes about 6 to 12 weeks.

## Discussion

The availability of TB confirmatory tests allowing a fast and conclusive detection of tuberculous mycobacteria in suspect animal tissues would be a great advantage in improving the efficiency of TB eradication programs and in decreasing the associated economic burden. Improvements in specificity, sensitivity and detection limit of diagnostic assays are usually introduced by molecular approaches. Nevertheless, only a few diagnostic PCR-based assays have been described for detecting MTC members directly in animal specimens, usually yielding limited sensitivities when compared to the reference bacteriological culture [8-12,15,19]. These moderate sensitivities may well be linked to the increased complexity for disrupting and recovering genomic DNA from the tough mycobacterial cells and to the paucibacillary nature of many animal tissue lesions used for nucleic acids extraction [10]. The presence of amplification inhibitors in crude tissue extracts, namely of large amounts of co-extracted eukaryotic DNA, may represent an additional problem. An option that has been explored to enhance the sensitivity of PCR techniques involves the implementation of more effective DNA extraction and purification methods. Taylor and colleagues were able to increase the *M. bovis* PCR detection sensitivity in bovine tissue samples with visible lesions, from 70% to 91%, after the inclusion of an additional step of liquid nitrogen freeze-thaw cycles in the DNA extraction procedure [10]. The use of sequence capture or immunomagnetic separation approaches was also forecasted for recovering higher yields of mycobacterial DNA from samples [8,9,12,21]. Nevertheless, these DNA extraction approaches usually involve more experimental steps and expensive equipments and consumables. 

In this work, an alternative approach was evaluated for enhancing MTC detection sensitivities directly from fresh animal tissues. An adapted and optimized user-friendly DNA extraction protocol, mainly based in the use of simple commercially obtainable extraction kits, was coupled with an IS*6110*-targeted semi-nested real-time PCR assay that allows the direct detection of MTC members in animal tissue specimens with very high sensitivity, specificity and positive and negative predictive values, namely in bovine specimens. These two last parameters are dependent on the prevalence of the tested condition in the population under study, as well as on the sensitivity and specificity of the testing assays. The prevalence of MTC (mostly *M. bovis*) infected samples among all TB-suspect samples submitted to the reference laboratory during the 2002-2010 period (n = 6364) was approximately 40% (reviewed by Cunha and colleagues [27]), which compares well with the prevalence values found in this work and used for the computation of PPV and NPV. Therefore, the PPV and NPV parameters computed may be considered good indicators of the performance of our IS*6110*-targeted assay for assessing the presence of MTC in specimens collected from animals clinically suspected of having tuberculosis and submitted for confirmatory culture analysis in a major reference laboratory.

Eight DNA-positive amplification results were obtained from tissue samples from which *M. bovis* could not be cultured (Table 2). However, lesions compatible with TB were observed in these tissues during histological analysis (sample types V and VI) (Table 2). The culture of tissue samples for the isolation of *M. bovis* and of other MTC members, followed by molecular or biochemical identification procedures, is usually the gold-standard method to validate alternative diagnostic assays. However, it is known that bacteriological culture is slow and laborious and can yield ambiguous or false-negative results, e.g. due to the presence of non-viable mycobacteria, raising concerns about its effectiveness as comparison reference method [19,31]. The results of culture assays may be affected by several factors, such as the harsh processing and decontamination procedures of samples, which can also have a harmful effect on *M. bovis* viability, as well as the growth media and incubation conditions used and the constrained distribution of mycobacteria in tissues [32]. Therefore, although TB-like lesions identified by histopathology can be induced by other bacteria or mycobacteria, the positive amplification results suggest that MTC members were most probably associated with the observed lesions and that the PCR test is more sensitive than bacteriological culture for detecting these pathogens in animal tissues. Non-MTC mycobacteria such as *M. avium* and *M. scrofulaceum* were cultured from five of those eight DNA-positive tissue samples (Table 2). These non-MTC mycobacteria may potentially overgrow any *M. bovis* isolate present in the sample and mask its presence [19]. Regardless of some discrepancies, the kappa measure of agreement between bacteriological culture and the semi-nested PCR was estimated in 0.859. Although the criteria for judging kappa statistic are not completely objective nor universally accepted, this value may allow us to infer an "almost perfect" agreement between the two MTC detection methods [33].

The enhancement of the MTC detection rates using the semi-nested amplification assay need to be balanced against the associated increased risk of cross contamination of samples. Therefore, we should emphasize the need of working in a veterinary diagnosis laboratory harboring good practice standard conditions for molecular analysis, which include working in separate clean rooms and the use of positive and negative controls.

In a preliminary survey the testing of TB positive and negative tissue samples employed a standard real-time PCR assay (corresponding to the second step of the semi-nested approach). The comparison of this assay with the reference of bacteriological culture showed a diagnostic sensitivity of only 40% (data not shown). The inclusion of the first step of conventional IS*6110*-targeted PCR amplification, in a semi-nested design, allowed to increase the sensitivity of the assay to near 100%. Previous studies found no significant improvements in the detection of MTC members in animal tissue samples using nested PCR assays, including with real-time PCR formats [10,14,15]. Nevertheless, the performance of PCR detection systems is highly dependent on the efficiency of the primers and probes, even when using the same genomic targets such as the IS*6110* element [34]. The MTC-specific IS*6110*-targeted primers and probe used in this work were shown to be highly efficient for detecting tuberculous mycobacteria in animal tissue samples, although the isolates of the most relevant species, *M. bovis*, usually contain only one copy of this insertion sequence. It has recently been found that IS*6110*-like elements may be present in other non-MTC mycobacteria such as *M. smegmatis* [35]. However, the probe and respective flanking primers used show no relevant complementary regions with these IS*6110*-like nucleotide sequences (Figure 2).

The protocol for direct detection of MTC from fresh animal tissues, using an optimized DNA extraction and purification procedure coupled with a semi-nested real time PCR assay described in this work, was shown to be highly accurate and much faster than bacteriological culture, reducing the time for confirmatory TB diagnosis from several weeks to few hours, thus also potentially decreasing the arrest period of the suspect animal carcass and herd. The assay is amenable to future automation possibilities regarding both the DNA extraction and amplification steps. It may also allow the detection of MTC members when these pathogens become nonviable and non-cultivable or are overgrown by other less fastidious bacteria or mycobacteria also present in tissue samples. The use of a novel *β-actin* gene targeted probe, and respective flanking primers, with complementary targets in most mammal species, allowed to assess the presence of amplification inhibitors in the DNA extracts. Although the test is not able to distinguish between different members of the MTC, particularly *M. bovis*, the identification of any tuberculous mycobacteria infection in domestic or wildlife animals could have public health implications. 
